# Intelligent Ultrasonic Aspirator Controlled by Fiber-Optic Neoplasm Sensor Detecting 5-Aminolevulinic Acid-Derived Porphyrin Fluorescence

**DOI:** 10.3390/s25113412

**Published:** 2025-05-28

**Authors:** Yoshinaga Kajimoto, Hidefumi Ota, Masahiro Kameda, Naosuke Nonoguchi, Motomasa Furuse, Shinji Kawabata, Toshihiko Kuroiwa, Toshihiro Takami, Masahiko Wanibuchi

**Affiliations:** 1 Department of Neurosurgery, Osaka Medical Pharmaceutical University, Takatsuki-City 569-8686, Japan; naosuke.nonoguchi@ompu.ac.jp (N.N.); motomasa.furuse@ompu.ac.jp (M.F.); shinji.kawabata@ompu.ac.jp (S.K.); toshihiko.kuroiwa@ompu.ac.jp (T.K.); toshihiro.takami@ompu.ac.jp (T.T.); masahiko.wanibuchi@ompu.ac.jp (M.W.); 2 Stryker Japan, Tokyo 160-0023, Japan; hidefumi.ota@stryker.com

**Keywords:** 5-aminolevulinic acid (5-ALA), protoporphyrin IX fluorescence, glioblastoma, malignant glioma surgery, photodynamic diagnosis (PDD), surgical robotics, fiber-optic tumor sensor

## Abstract

The development of an intelligent ultrasonic aspirator controlled by a fiber-optic neoplasm sensor that detects 5-aminolevulinic acid-derived porphyrin fluorescence presents a significant advancement in glioma surgery. By leveraging the fluorescence phenomenon associated with 5-aminolevulinic acid in malignant neoplasms, this device integrates an excitation laser and a high-sensitivity photodiode into the tip of an ultrasonic aspirator handpiece. This setup allows for real-time tumor fluorescence detection, which in turn modulates the aspirator’s power based on fluorescence intensity. Preliminary testing demonstrated high sensitivity, with the device capable of differentiating between weak, strong, and no fluorescence. The sensor sensitivity was comparable to human visual perception, enabling effective tumor differentiation. Tumors with strong fluorescence were effectively crushed, while the aspirator ceased operation in non-fluorescent areas, enabling precise tissue resection. Furthermore, the device functioned efficiently in bright surgical environments and was designed to maintain a clean sensor tip through constant saline irrigation. The system was successfully applied in a surgical case of recurrent glioblastoma, selectively removing tumor tissue while preserving surrounding brain tissue. This innovative approach shows promise for safer, more efficient glioma surgeries and may pave the way for sensor-based robotic surgical systems integrated with navigation technologies.

## 1. Introduction

Accurately determining the extent of neoplasms during surgery is often challenging, particularly for malignant tumors with invasive characteristics. Tumor invasion frequently blurs the boundary between neoplastic and normal tissue. This issue is especially pronounced in malignant gliomas, among the most invasive tumor types, where the margins are indistinct. Surgeons typically rely on empirical assessments of various tumor characteristics, such as color, hardness, and bleeding tendency, to differentiate tumors from normal tissue. However, these judgments are often subjective and prone to error.

Photodynamic diagnosis (PDD) using 5-aminolevulinic acid (5-ALA) has proven to be an effective method for visualizing various malignant neoplasms, including bladder cancer [1], prostate cancer [2], bronchogenic cancer [3], esophageal cancer, skin cancer, and brain tumors [4,5,6,7,8,9,10]. This technique enhances the surgeon’s ability to assess tumor extension. In neurosurgery, the use of 5-ALA PDD in malignant glioma surgery has been shown to improve resection rates and extend progression-free survival [6,7,8,9,10]. The fluorescence of 5-ALA-induced protoporphyrin IX (PpIX) is tumor-specific and sufficiently strong to be visible to the naked eye, providing a critical visual aid during surgery.

Recent advances in robotics technology have enabled the development of master–slave surgical robotic systems. However, autonomous and intelligent surgical robotic systems capable of treating neoplasms have yet to be realized. A key requirement for such systems is the incorporation of advanced sensing technologies that can detect neoplasms with high specificity and temporal resolution. To date, no sensors meeting these criteria have been developed.

A fiber-optic tumor sensor leveraging 5-ALA-induced fluorescence has been introduced to specifically detect tumor-derived fluorescence [11]. In this study, we integrated this sensor into an ultrasonic aspirator, creating a tumor sensor-controlled intelligent ultrasonic aspirator. This system is designed to automatically and selectively resect malignant brain tumors while minimizing damage to surrounding normal tissue. Here, we describe the mechanism and functionality of this novel tumor sensor and the intelligent ultrasonic aspirator, as well as their potential clinical applications.

## 2. Materials and Methods

### 2.1. Fiber Optic Neoplasm Sensor

**Figure 1:**
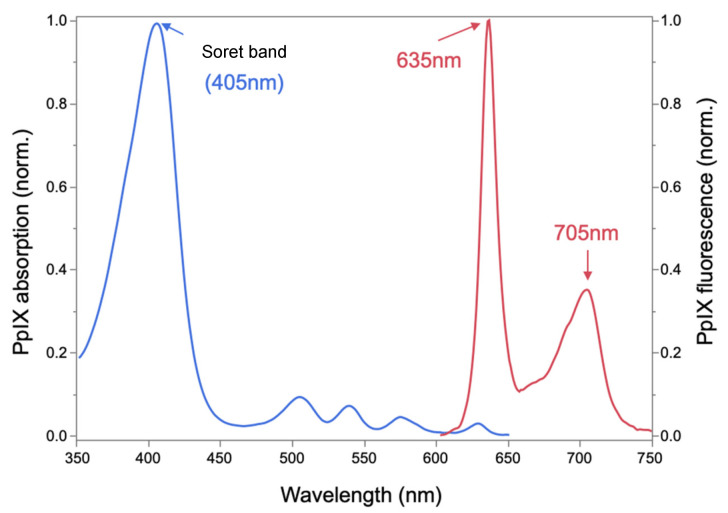
Absorption and fluorescence spectra of PpIX.

We designed a malignant neoplasm sensor to detect the fluorescence of protoporphyrin IX (PpIX), which is produced from 5-ALA in malignant neoplasm cells via the intracellular heme biosynthesis pathway. The wavelength at which PpIX is most efficiently excited is 405 nm in the Soret band (Figure 1). Therefore, we employed a violet laser (λ = 405 nm, 5 mW) as the excitation light source. The laser light was focused and guided to one end of a plastic optical fiber (φ = 1 mm). Excitation light emitted from the other end of the optical fiber to the surgical field excites PpIX in the tumor, and a portion of the fluorescence returns through the optical fiber. The returned light contains excitation light, illumination light, tissue autofluorescence, and PpIX fluorescence.

**Figure 2:**
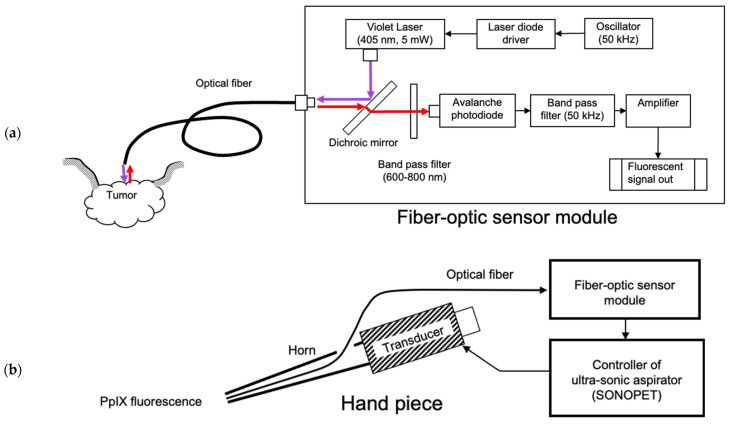
Diagram of the device: (**a**) A 405 nm, 5 mW violet laser beam modulated at 50 kHz is guided into an optical fiber via a dichroic mirror. The excitation light emitted from the optical fiber tip irradiates the surgical field. Red fluorescence emitted from PpIX within the tumor is collected through the optical fiber and guided into the device. A 500 nm dichroic mirror and a 600–800 nm band-pass filter are used to optically eliminate non-fluorescent light. Additionally, a 50 kHz electrical band-pass filter suppresses ambient light interference. These optical and electrical filtering processes enable the detection of fluorescence exclusively from PpIX. (**b**) The optical fiber is coaxially inserted into the handpiece of the ultrasonic aspirator. The PpIX signal output from the sensor module is connected to the ultrasonic aspirator’s controller, which adjusts the intensity of tissue fragmentation according to the signal strength.

PpIX fluorescence is faint compared to the intense illumination light from the operating microscope. To distinguish the fluorescence signal from other light, we used a two-stage bandpass filter system (Figure 2a). The first stage employed an optical bandpass filter, which transmits light with wavelengths between 600 nm and 800 nm. Since the peak wavelengths of PpIX are 635 nm and 705 nm, this filter efficiently transmits PpIX fluorescence while removing most illumination light and green autofluorescence.

The second stage utilized a 50 kHz electrical bandpass filter. Highly sensitive avalanche photodiodes converted the filtered light into electrical signals. The violet laser for fluorescence excitation was amplitude-modulated at 50 kHz, causing the PpIX fluorescence to oscillate at the same frequency. The 50 kHz bandpass filter selectively output only the oscillating PpIX fluorescence signal. The detected fluorescence intensity was displayed on an LED (Light-Emitting Diode) indicator on the front panel in 10 increments.

### 2.2. Control System for Intelligent Ultrasonic Aspirator

We modified the ultrasonic aspirator (SONOPET UST-2001, Stryker, Kalamazoo, MI, USA) so that its sensor output could control the vibration intensity of the ultrasonic aspirator (Figure 2b). This modified device, named the “Intelligent Sonopet”, was designed with a response time of 10 milliseconds, significantly faster than the human visual reaction time (170 ms).

**Figure 3:**
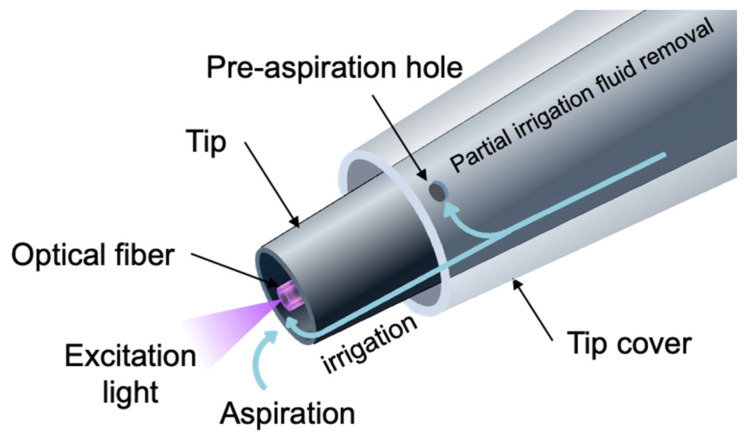
Irrigation fluid flows out from the tip cover, irrigates the surgical field, and is subsequently aspirated along with fragmented tissue and blood. The tip of the optical fiber is continuously kept clean by the irrigation fluid.

The fiber-optic sensor was inserted coaxially into the handpiece cylinder via an aspiration channel (Figure 2b and Figure 3). The sensor tip was aligned with the aspirator tip, providing three advantages:

1. Direct contact between the sensor tip and the area of interest, such as a tumor.
2. Minimal gap between the sensor and aspirator tips.
3. Effective removal of obstructions like blood via saline irrigation and aspiration.

The other end of the optical fiber was connected to a fiber-optic sensor module (Figure 3).

### 2.3. Comparison of Fiber-Optic Neoplasm Sensor and Human Visual Assessment of PpIX Fluorescence

**Figure 4:**
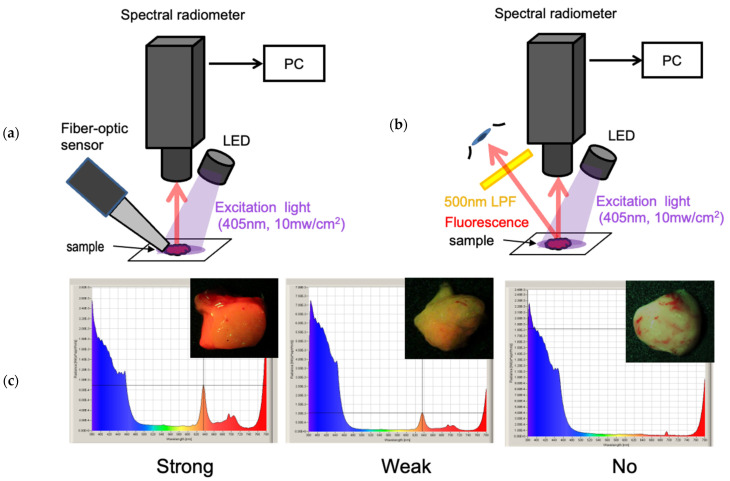
Comparison of sensitivity characteristics of the fiber-optic neoplasm sensor and human visual assessment of PpIX fluorescence using a spectroradiometer: The sensitivity characteristics of both methods can be quantified by measuring the spectral fluorescence intensity of PpIX fluorescence. The comparison of both provides insights into the differences in fluorescence sensitivity characteristics. (**a**) The fiber-optic sensor system is applied to 11 resected tumor samples for measurement. The fluorescence intensity is indicated by an LED indicator on a scale from 0 to 10. Simultaneously, the PpIX fluorescence intensity of the tumor is measured using a spectral radiometer. The excitation light intensity of 10 mW/cm^2^ corresponds to the typical excitation light intensity of a fluorescence surgical microscope. (**b**) A different set of 43 tumor samples is observed with the naked eye through a 500 nm low-pass filter (LPF). The fluorescence intensity is assessed as strong, weak, or no fluorescence. Simultaneously, the PpIX fluorescence of the tumor is measured using a spectral radiometer. (**c**) Spectral spectrum and visual fluorescence images of the tumor. Strong fluorescence shows a prominent peak at 635 nm, while no fluorescence shows no such peak. Weak fluorescence is observed as an intermediate intensity.

We evaluated the sensitivity of the fiber-optic neoplasm sensor compared to human visual assessment of PpIX fluorescence using spectral radiance measurements (Figure 4a,b).

First, we analyzed the relationship between the detection levels of the fiber-optic neoplasm sensor and spectral radiance using 11 excised glioblastoma tumor specimens (Figure 4a). The excitation light intensity at the sample surface was 10 mW/cm^2^, simulating typical fluorescence operating microscope conditions. The detection level of the PpIX fluorescence was displayed on the sensor’s front panel on a scale of 0 to 10. Spectral radiance at 635 nm was quantified using a Spectroradiometer (Specbos1201 with focusing optics, JETI Technische Instrumente GmbH, Jena, Germany).

Second, we evaluated the relationship between the visible fluorescence intensity and spectral radiance using 43 excised glioblastoma tumor specimens (Figure 4b,c). Using a 405 nm LED excitation source, we assessed PpIX fluorescence intensity visually and measured spectral radiance at 635 nm. Visual assessments were performed through a 500 nm low-pass filter and rated on a three-point scale: strong, weak, or none. These experiments allowed us to estimate the correlation between visible fluorescence intensity and sensor detection levels.

### 2.4. Application of the Intelligent Ultrasonic Aspirator on Resected Tumor Masses

Before clinical application, we assessed the intelligent ultrasonic aspirator on resected tumor masses under the following conditions:

1. Detection of PpIX fluorescence by the fiber-optic sensor under microscopic illumination.
2. Deactivation of the ultrasonic aspirator in normal brain tissue.
3. Real-time response and time delay of the ultrasonic aspirator.

We recorded PpIX fluorescence detection by the fiber-optic sensor in small tumor masses with strong fluorescence under operating microscope illumination. Additionally, we tested the aspirator’s performance on resected temporal lobe tissue containing both normal brain and glioblastoma.

### 2.5. Preliminary Clinical Application in Malignant Glioma Resection

The clinical application protocol of the intelligent ultrasonic aspirator was approved by the ethics committee of Osaka Medical College (No. 42, 309). Written informed consent was obtained from all patients and their families.

We applied the system during surgery on a patient with recurrent glioblastoma in the right frontal lobe. The study aimed to verify the following:

1. Whether the system could detect tumors under white light and allow appropriate operation of the ultrasonic aspirator.
2. Whether the system could halt operation at the tumor-brain border to protect surrounding normal brain tissue.

## 3. Results

### 3.1. Comparison of Sensitivity to PpIX Fluorescence Between the Fiber-Optic Sensor and the Naked Eye

**Figure 5:**
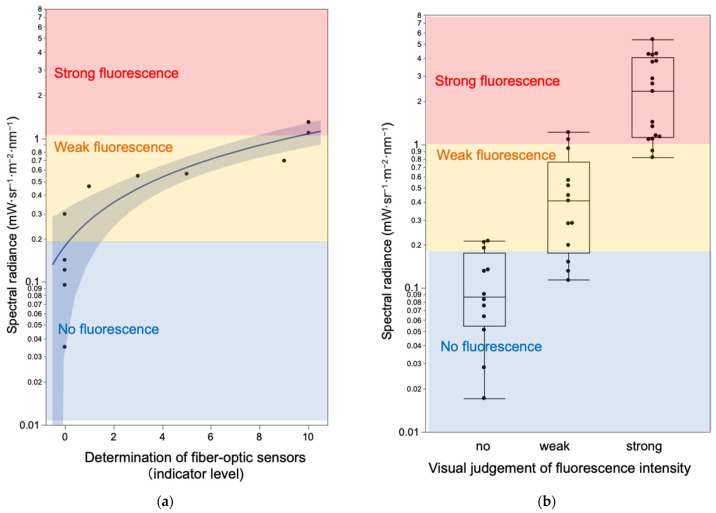
Sensitivity characteristics of the fiber-optic sensor (**a**) and the naked eye (**b**). (**a**) In the strong fluorescent region, the fluorescence indicator showed a value of 10, while in the no fluorescence region, it showed a value of 0. In the weak fluorescence region, the values ranged from 1 to 9. These characteristics were almost identical to the fluorescence detection characteristics of the naked eye (**b**).

During a craniotomy, the surgeon uses a microscope with a fluorescent filter to check the presence or absence and even intensity of PpIX fluorescence during the operation, while adjusting the power of the ultrasound aspirator to remove the tumor. Since this decision-making process of the surgeon must eventually be integrated into the ultrasonic aspirator as a system based on the sensor, we first compared the sensitivity of the fiber-optic sensor with that of the naked eye to PpIX fluorescence. The detection limit of the sensor module, corresponding to an indicator level of 1, was determined to be 0.46 mW·sr^−1^·m^−2^·nm^−1^. This fluorescence intensity matches the threshold for faint fluorescence as perceived by the naked eye (Figure 5). The indicator level reached its maximum value of 10 at 1.1 mW·sr^−1^·m^−2^·nm^−1^, which corresponds to strong fluorescence (Figure 5a).

Figure 5a illustrates the relationship between the three fluorescence intensity levels assessed visually (none, weak, and strong) and the spectral radiance of the PpIX fluorescence peak at 635 nm. The boundary between no fluorescence and weak fluorescence was 0.2 mW·sr^−1^·m^−2^·nm^−1^, while the boundary between weak and strong fluorescence was 1.1 mW·sr^−1^·m^−2^·nm^−1^. These results demonstrate that the sensitivity of the fiber-optic sensor aligns well with human perception of fluorescence intensity.

### 3.2. Experimental Application on Resected Tumor Masses Under an Operating Microscope

**Figure 6:**
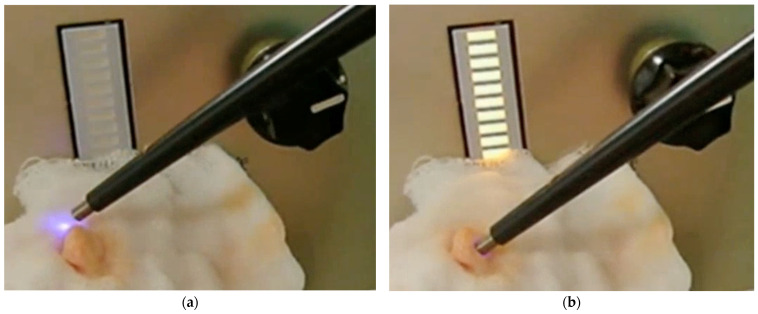
Operational status of the intelligent ultrasonic aspiration device in tumor resection specimens: (**a**) When the tumor is not in contact with the tip of the handpiece, the output of the indicator is zero. A purple excitation light is visible from the tip of the handpiece. (**b**) When the tip of the handpiece contacts with the tumor, the indicator shows level 10.

The intelligent ultrasonic aspirator reliably detected PpIX fluorescence in small tumors under bright illumination from an operating microscope, as indicated in real-time by the front panel. When the handpiece tip contacted the tumor, the sensor detected fluorescence instantaneously, with no observable response delay (Figure 6, Movie S1). In resected tissue containing both normal brain and tumor regions, the device selectively fragmented and aspirated tumor tissue in the PpIX fluorescent regions (Movie S2). In contrast, when the handpiece contacted adjacent non-fluorescent brain tissue, the ultrasonic aspirator remained inactive, preserving the normal brain. These results confirmed that the system is sufficiently sensitive to detect PpIX fluorescence in tumors and selectively remove the fluorescent regions under an operating microscope in real time.

### 3.3. Clinical Application of the Intelligent Ultrasonic Aspirator

**Figure 7:**
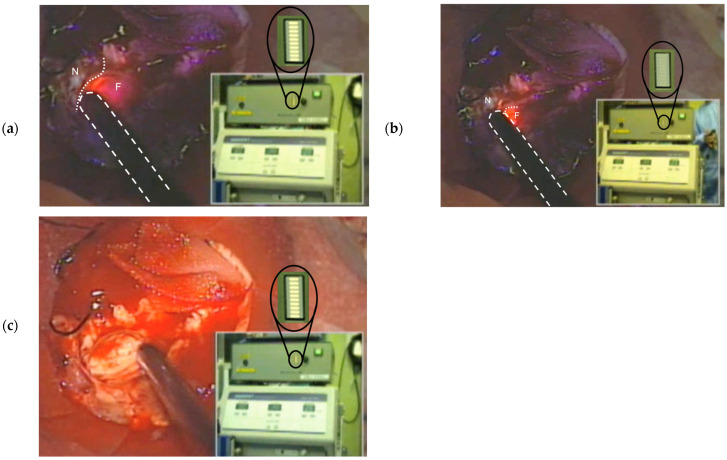
Application of the intelligent ultrasonic aspirator in recurrent glioblastoma resection: (**a**) When the tip of the handpiece contacts the strong fluorescent tumor in fluorescence mode, the intelligent ultrasonic aspirator detects it with indicator level 10 and proceeds with tumor fragmentation and aspiration. (**b**) When the tip of the handpiece contacts the non-tumor area with no fluorescence, the indicator level becomes 0, and tumor fragmentation stops. (**c**) Even in white light mode, the intelligent ultrasonic aspirator operates selectively on the tumor without being affected by the strong microscope illumination. The white dashed line represents the outline of the handpiece. The white solid line marks the boundary between the fluorescent (F) and non-fluorescent (N) regions.

The intelligent ultrasonic aspirator was clinically applied in a case of recurrent glioblastoma. Tumor resection was initially performed under fluorescent-mode illumination using an operating microscope (Figure 7, Movie S3). The device accurately detected areas of strong tumor fluorescence and facilitated selective tissue fragmentation and aspiration (Figure 7a). The fiber-optic sensor tip remained operational even when blood obscured the surgical field, thanks to continuous irrigation and aspiration. As tumor removal progressed and fluorescence diminished, the indicator disappeared, and the aspirator ceased fragmentation activity (Figure 7b). The system demonstrated no perceptible response delay throughout the procedure.

Subsequently, the procedure was performed under white light mode illumination with bright white light. The intelligent ultrasonic aspirator functioned equivalently well under these conditions (Figure 7c). By integrating the intelligent aspirator with fluorescence-guided surgery, gross-total tumor resection was achieved, with the patient experiencing no postoperative neurological deficits.

## 4. Discussion

### 4.1. Intelligent Ultrasonic Aspirator for Microscopic Tumor Resection

In this study, we developed a fiber-optic neoplasm sensor capable of detecting protoporphyrin IX (PpIX) fluorescence induced by 5-aminolevulinic acid (5-ALA). Additionally, we integrated this sensor into an ultrasonic aspirator, enabling the control of ultrasonic vibration output based on fluorescence intensity. Our results demonstrate that the device’s sensitivity to PpIX fluorescence is comparable to that of human detection. The intelligent ultrasonic aspirator operated at maximum vibration within tumor regions exhibiting strong fluorescence, while halting ultrasonic activity in non-fluorescent normal tissues. Consequently, this device acts as a safety mechanism, preserving non-fluorescent normal tissues without causing physical damage.

Two primary challenges were anticipated in the implementation of a fiber-optic sensing system for PpIX fluorescence: (1) the interference from strong background illumination during microscopy and (2) light transmission obstruction at the fiber-optic sensor tip due to blood. We demonstrated that the sensor reliably controls the ultrasonic aspirator despite these challenges, ensuring stable operation under high-intensity illumination and during sensor occlusion by blood.

### 4.2. Complementary Role of the Intelligent Ultrasonic Aspirator in Fluorescence-Guided Surgery

The intelligent ultrasonic aspirator operates effectively under white-light conditions, enabling fluorescence-guided surgery without requiring exclusive use of fluorescence mode. A key limitation of fluorescence-guided surgery is the difficulty in identifying normal structures due to the darkened surgical field in fluorescence mode. This device, when used in conjunction with a fluorescence microscope, addresses this drawback.

Fluorescence-guided tumor resection is less effective in four specific scenarios: (1) deep surgical fields with limited illumination, (2) tumors located on walls parallel to the microscope’s optical axis, (3) tumors prone to heavy bleeding, where the resection surface is obscured by blood, and (4) deep resection surfaces shadowed by tumor masses. The intelligent ultrasonic aspirator overcomes these limitations, complementing fluorescence-guided surgery in challenging conditions.

### 4.3. Mitigation of Intensive Microscope Illumination Effects

To address the issue of strong background illumination from the microscope, we implemented a dual-filtering approach comprising optical and electrical bandpass filters. This study confirms the effectiveness of these measures, demonstrating reliable device performance during tumor resection even under intense illumination.

By modulating the excitation light at 50 kHz, PpIX fluorescence is induced to flicker at the same frequency, while background light remains unaffected. The 50 kHz electrical bandpass filter effectively isolates the PpIX fluorescence signal from the background illumination. Additionally, since red spectral components in microscope illumination overlap with PpIX fluorescence, an optical pre-filter system—consisting of a 500 nm dichroic mirror and a 600–800 nm bandpass filter—was employed to prevent sensor saturation. The successful operation of the system under these conditions validates the robustness of the dual-filtering strategy.

### 4.4. Overcoming Light Blockage by Blood at the Sensor Tip

To resolve the issue of blood obstructing light transmission at the fiber-optic sensor tip, we employed continuous irrigation at the handpiece tip. This constant flow of irrigation fluid ensures the fiber tip remains clean by washing away blood and other obstructions. The device demonstrated reliable performance in blood-covered surgical fields, effectively maintaining light transmission throughout the procedure.

### 4.5. Time Resolution and System Responsiveness

For any sensor-based control system, a high time resolution and rapid responsiveness are critical for safe and stable operation. Our system employs an open-loop control mechanism, free from time delays. The sensing system has a time resolution of 10 ms, translating to an overall system response time of 10 ms—approximately 20 times faster than the human visual response time of 180–220 ms [12].

During tumor resection, if the ultrasonic aspirator tip encounters a blood vessel or normal brain tissue, immediate cessation of ultrasonic activity is essential to prevent damage. The superior response time of this device enhances safety by minimizing the risk of inadvertent harm to healthy tissues.

### 4.6. Application to Alternative Fluorescent Labeling Agents

This system can be adapted for use with fluorescent labeling agents other than PpIX by adjusting the excitation and bandpass filter wavelengths to match the spectral properties of the chosen fluorophore. For agents with weaker fluorescence signals, enhancements such as increased laser power, improved optical sensor integration, and heightened sensitivity of avalanche photodiodes can be implemented.

### 4.7. Towards Sensor-Controlled Robotic Surgical Systems

The intelligent ultrasonic aspirator represents a pioneering step towards sensor-based autonomous robotic surgical systems. Unlike current master–slave robotic systems, such as the da Vinci system [13,14], which rely on manual surgeon control, this device incorporates autonomous manipulation capabilities. The high tumor specificity of 5-ALA-induced PpIX fluorescence establishes our fiber-optic tumor sensor as a reliable foundation for future sensor-based surgical robotics.

### 4.8. Standalone Use and Integration with Surgical Navigation Systems

As demonstrated, this system can be used independently to address situations where fluorescence visibility is challenging during conventional fluorescence-guided surgery. It eliminates the need to switch between fluorescence and white-light modes of the fluorescence microscope, streamlining the surgical workflow and reducing the risk of inadvertently damaging normal tissues. Consequently, it has the potential to make malignant glioma surgeries faster and more precise.

Furthermore, integrating this system with a surgical navigation system would allow for fluorescence intensity data to be plotted on a navigation map, enabling surgeons to delineate tumor resection boundaries with greater accuracy. This integration could also facilitate intraoperative brain shift correction. The combination of the intelligent ultrasonic aspirator with navigation technology holds promising potential for the development of advanced surgical support systems for brain tumor resection.

## 5. Conclusions

We developed an intelligent ultrasonic aspirator with a fiber-optic neoplasm sensor detecting 5-ALA-induced fluorescence, enabling precise glioma resection. The sensor demonstrated high sensitivity, detecting fluorescence at levels comparable to visual perception, even under surgical lighting and blood obscuration. With a rapid 10 ms response, the system selectively halts aspiration upon detecting non-fluorescent tissue, enhancing safety and accuracy. This technology represents a significant step toward fluorescence-guided, precision surgery and future integration with robotic and navigation systems.

## Data Availability

Relevant data are available upon reasonable request to the corresponding author.

## Supplementary Materials

The following supporting information can be downloaded at: https://www.mdpi.com/article/10.3390/s25113412/s1.

## Abbreviations

The following abbreviations are used in this manuscript:

PDD	Photodynamic diagnosis
5-ALA	5-aminolevulinic acid
PpIX	protoporphyrin IX
LED	Light Emitting Diode

## References

[1] Kriegmair M., Baumgartner R., Knüchel R., Stepp H., Hofstädter F., Hofstetter A. (1996). Detection of early bladder cancer by 5-aminolevulinic acid induced porphyrin fluorescence. J. Urol..

[2] Zaak D., Sroka R., Khoder W., Adam C., Tritschler S., Karl A., Reich O., Knuechel R., Baumgartner R., Tilki D. (2008). Photodynamic diagnosis of prostate cancer using 5-aminolevulinic acid—First clinical experiences. Urology.

[3] Huber R.M., Gamarra F., Hautmann H., Häußinger K., Wagner S., Castro M., Baumgartner R. (1999). 5-Aminolaevulinic acid (ALA) for the fluorescence detection of bronchial tumors. Diagn. Ther. Endosc..

[4] Kajimoto Y., Kuroiwa T., Miyatake S., Ichioka T., Miyashita M., Tanaka H., Tsuji M. (2007). Use of 5-aminolevulinic acid in fluorescence-guided resection of meningioma with high risk of recurrence: Case report. J. Neurosurg..

[5] Miyatake S., Kuroiwa T., Kajimoto Y., Miyashita M., Tanaka H., Tsuji M. (2007). Fluorescence of non-neoplastic, magnetic resonance imaging-enhancing tissue by 5-aminolevulinic acid: Case report. Neurosurgery.

[6] Pichlmeier U., Bink A., Schackert G., Stummer W. (2008). Resection and survival in glioblastoma multiforme: An RTOG recursive partitioning analysis of ALA study patients. Neuro Oncol..

[7] Stummer W., Novotny A., Stepp H., Goetz C., Bise K., Reulen H.J. (2000). Fluorescence-guided resection of glioblastoma multiforme by using 5-aminolevulinic acid–induced porphyrins: A prospective study in 52 consecutive patients. J. Neurosurg..

[8] Stummer W., Pichlmeier U., Meinel T., Wiestler O.D., Zanella F., Reulen H.J., ALA-Glioma Study Group (2006). Fluorescence-guided surgery with 5-aminolevulinic acid for resection of malignant glioma: A randomized controlled multicentre phase III trial. Lancet Oncol..

[9] Stummer W., Stocker S., Wagner S., Stepp H., Fritsch C., Goetz C., Goetz A.E., Kiefmann R., Reulen H.J. (1998). Intraoperative detection of malignant gliomas by 5-aminolevulinic acid-induced porphyrin fluorescence. Neurosurgery.

[10] Stummer W., Reulen H.J., Meinel T., Pichlmeier U., Schumacher W., Tonn J.C., Rohde V., Oppel F., Turowski B., Woiciechowsky C. (2008). Extent of resection and survival in glioblastoma multiforme: Identification of and adjustment for bias. Neurosurgery.

[11] Kajimoto Y., Miyatake S., Kuroiwa T. (2004). Fiber-optic spectroscopic detection of neoplasm by intraoperative fluorescence labeling. Int. Congr. Ser..

[12] Brebner J.T., Welford A.T., Welford A.T. (1980). Introduction: An historical background sketch. Reaction Times.

[13] Jung M., Morel P., Buehler L., Buchs N.C., Hagen M.E. (2015). Robotic general surgery: Current practice, evidence, and perspective. Langenbeck’s Arch. Surg..

[14] Peters B.S., Armijo P.R., Krause C., Choudhury S.A., Oleynikov D. (2018). Review of emerging surgical robotic technology. Surg. Endosc..

